# The impact of depression on language function in individuals with Alzheimer’s disease: a pre/post-treatment design

**DOI:** 10.1186/s12991-023-00433-6

**Published:** 2023-02-03

**Authors:** Kyung Hee Yoon, Yoo Sun Moon, Do Hoon Kim

**Affiliations:** 1 Department of Psychiatry, Chuncheon Sacred Heart Hospital, Hallym University College of Medicine, 77 Sakju-Ro, Chuncheon, 24253 Republic of Korea; 2 Mind-Neuromodulation Laboratory, Chuncheon Sacred Heart Hospital, Hallym University College of Medicine, 77 Sakju-Ro, Chuncheon, 24253 Republic of Korea

**Keywords:** Alzheimer’s disease, Depression, Cognitive function

## Abstract

**Background:**

It is uncertain whether depression might affect cognitive function in Alzheimer’s disease (AD). Most of studies on the effect of depression treatment on cognitive function in AD were briefly evaluated by Mini-Mental State Examination (MMSE). MMSE is poor sensitive to detect cognitive change. This study examined the cognitive response to depression treatment in AD via multi-domain assessment. In addition, we explored whether effect of depression treatment in AD is different those of late-life depression (LLD).

**Methods:**

This study include AD patients with depression (AD + D) and without depression (AD − D), LLD patients (LLD), and healthy controls (HC). The patients were treated according to their diagnosis for 16 weeks: acetylcholinesterase inhibitors (AChEIs) and selective serotonin reuptake inhibitors (SSRIs) for AD + D, AChEIs for AD − D, and SSRIs for LLD. The cognitive changes from pre- to post-treatment were compared between AD + D and AD − D or LLD and HC. An independent sample *t* test was performed to compare the degree of change between the groups. Paired *t* tests were used to determine cognitive function changes in each depression treatment responder group.

**Results:**

At baseline, AD + D had more impairment in language function compared to AD − D, and LLD had greater deficit in executive function than HC. After depression treatment, more impaired cognitive domains at baseline were improved in AD + D and LLD, respectively. Moreover, AD + D showed an improvement in the global cognitive function (MMSE).

**Conclusions:**

Results indicated that language function was influenced by depression in AD, which is first evidence for specific cognitive domain related to depression in AD. Our finding indicates that depression could negatively impact cognitive function, and depression treatment may have beneficial cognitive effect in both AD and LLD. This study suggests the importance of early detection and treatment of depression in AD and LLD.

*Trial registration* Clinical Research Information Service, CRIS, ID#: KCT0004041, Registered 5 June 2019, retrospectively registered after first patient enrollment date (4 March 2014) https://cris.nih.go.kr/cris/search/detailSearch.do?seq=14140&status=5&seq_group=14140&search_page=M.

## Background

Depression is one of the most common comorbidities in patients with Alzheimer's Disease (AD). It has been estimated that 10–20% of AD patients have major depression, and an additional 40–50% of patients experience depressive symptoms [50]. The comorbidity of depression in AD patients is associated with greater impairment in activities of daily living (ADL) [61], worse quality of life (QOL) [22], increased behavioral disturbances [41], and suicidal risk [53]. Previous studies have suggested that depression in AD contributes to poor prognosis of AD [3, 48, 60].

Several cross-sectional comparisons indicate that AD patients with depression (AD + D) have more cognitive impairment than AD patients without depression (AD − D) [3, 66]. In contrast, other studies reported no differences in cognitive function between AD + D and AD − D [18, 21, 25, 34, 38]. An experimental study design is required to examine the casual relationships between AD, depression, and cognitive function, but few such studies have been conducted [47].

A clinical review of the literature and meta-analyses several studies, which have evaluated the impact of antidepressant treatment on cognitive function among AD patients with depression, suggested conflicting results [36, 47].

Some studies have shown an improvement in global cognitive function after use of serotonin reuptake inhibitors (SSRIs; citalopram, escitalopram, fluoxetine, and paroxetine) or tricyclic antidepressants (TCAs; amitriptyline) medication in AD patients with depression [52, 63]. However, it is not known which specific cognitive domains are improved from these studies, because cognitive function was only assessed globally with the Mini-Mental State Examination (MMSE). MMSE is used to measure global cognitive function as a brief and simple screening tool, but its use has not been extended to encompass diverse cognitive domains [7].

In contrast, several studies have reported that cognitive function was unchanged despite a reduction in depressive symptoms or recovery after SSRIs (sertraline) [40, 43] or TCAs (imipramine) medication treatment [49] in AD + D. In two of these studies, cognitive function was also evaluated broadly using the MMSE [40, 49]. MMSE is not an appropriate measure to detect cognitive alteration, because it has intrinsic limitations as tool for tracking cognitive changes [51]. Another study using a number of cognitive measurements including MMSE, the Alzheimer’s Disease Assessment Scale–Cognitive Subscale, Letter Fluency, Backward Digit Span, Symbol Digit Modalities Test, and Finger Tapping Test, found that sertraline treatment did not have a significant effect on any cognitive functions [43]. As a result, specific cognitive domains related to depression in AD are yet unknown. Because most previous studies focused on examining the efficacy of antidepressants as a primary outcome, cognitive function was only briefly evaluated using the MMSE. Thus, to clarify whether depression treatment in AD affects cognitive function, it is necessary to conduct a more detailed evaluation including a variety of cognitive domains.

Unlike depression in AD, it has been widely suggested that late-life depression (LLD) without dementia could induce cognitive impairment in the elderly [64, 67, 69]. Cognitive deficits in attention, executive function, and information processing speed are commonly noted in LLD [3, 13, 32, 67]. According to meta-analysis, the use of antidepressants to treat LLD has a moderate but not robust effect [44], while the efficacy of antidepressants for treatment of depression in AD has not been demonstrated [45, 47]. The clinical reviews of literature and meta-analysis, which included randomized controlled trials (RCTs) for efficacy of antidepressants vs. placebo with AD, identified only partial or no clinical benefits in treating depression in AD [36, 39, 45, 47]. As a result, it has been hypothesized that pathogenic mechanisms of depression in AD may be fundamentally different from those without AD [9, 11, 17]. Therefore, the effect of depressive treatment on cognitive function in AD is likely different from those in LLD.

As mentioned above, in most previous studies, cognitive response to depression treatment in AD was crudely measured by the MMSE, which showed conflicting results. To evaluate the influence of depression treatment on cognitive function more sensitively, diverse cognitive domains must be investigated using a comprehensive neuropsychological test battery.

Therefore, we investigated cognitive alterations between AD + D and AD − D via multi-domain assessment, including language function, memory, constructional praxis, and executive function, as well as global cognitive function in the MMSE, along with depression treatment. The effects of antidepressant treatment on each cognitive domain were also examined. In addition, we examined whether the effect of depression treatment in AD is different from that in LLD.

Specifically, this study examined the following by multi-domain assessment: (i) whether cognitive function is different according to the presence or absence of depression in AD group or elderly without dementia group and (ii) whether cognitive function changes after depression treatment in AD + D group or LLD group, and, if so, which cognitive domains are related to depression.

## Methods

### Participants

The participants were recruited from the Dementia Clinic of Chuncheon Sacred Heart Hospital. The inclusion criteria for the AD patient group (AD + D or AD − D) were as follows: (a) diagnosis of AD: met the criteria for AD via the Diagnostic and Statistical Manual of Mental Disorders, Fifth Edition (DSM-5) [4]; and neuropsychological tests that used the Korean version of the Consortium to Establish a Registry for Alzheimer’s Disease Assessment Packet (CERAD-K) [35]; (b) the diagnosis of depression for AD patients: three or more symptoms on the Olin Diagnosis Criteria for Depression in Alzheimer’s disease [46]; and (c) a score of 0.5–2 on the Clinical Dementia Rating (CDR) Scale [26].

Moreover, depression in the elderly without dementia was assessed according to the Diagnostic and Statistical Manual of Mental Disorders, Fifth Edition (DSM-5) [5].

The patients who met any of the following criteria at screening were excluded: (a) a history of taking antidepressants within 4 weeks; (b) treatment with memantine medication for at least 4 weeks; and (c) Parkinson's disease, stroke, brain tumor, or normal brain pressure hydrocephalus.

Eligible participants were enrolled 2 years (March 2014 to June 2016). A total of 95 participants were included in this study. Participants consisted of patients with newly diagnosed AD and LLD, as well as healthy controls with non-demented and non-depressed volunteers: 29 AD patients with depression (AD + D), 36 AD patients without depression (AD − D), 15 patients with late-life depression (LLD), and 15 healthy controls (HC) were included in the final sample.

All participants and their caregivers provided written informed consent. The Ethics and Medical Research Committee of Chuncheon Sacred Heart Hospital approved the study protocol.

## Procedures

All participants were scheduled at baseline and at 4, 8, and 16 weeks. Depressive symptoms were rated at every visit, while cognitive function, overall functioning, and other psychosocial factors were examined at the baseline and final visits.

Clinical groups received treatment with Acetylcholinesterase inhibitors (AChEIs; donepezil) (for AD) and/or selective serotonin reuptake inhibitors (SSRIs; escitalopram) (for depression) according to diagnosis during the 16 weeks: AChEIs and SSRIs for AD + D, AChEI for AD − D, and SSRIs for LLD.

## Assessments

### Depressive symptoms evaluations

Depressive symptoms in AD + D and LLD were objectively assessed by the Cornell Scale for Depression in Dementia (CSDD) [1] and the 17-item Hamilton Rating Scale for Depression (HAMD) [68], respectively. Because depression in AD has a different presentation [36], the dementia-specified CSDD was used to evaluate depression in AD patients. The CSDD is a validated clinician-administered instrument specifically designed to rate the symptoms of depression in dementia patients [1]. The HAMD is a widely used clinician-rated scale of depression in older adults; however, its validity has not been established for late-life depression[37]. Subjective depressive symptoms in both groups were measured using the Short Geriatric Depression Scale-Korean version (SGDS-K) [6]. The GDS is a self-report inventory specifically designed to measure depression among older adult population [8]. Higher scores reflect more severe feelings of depression. Treatment response was defined as a ≥ 50% reduction in CSDD for AD + D and HAMD score for LLD.

### Cognitive function evaluations

Cognitive function was assessed using several subtests from CERAD-K neuropsychological assessments and the Seoul Neuropsychological Screening Battery (SNSB) [28]. The neuropsychological measurement comprised nine subtests from CERAD-K and seven subtests from the SNSB. The subtests from CERAD-K; the Mini-Mental State Examination-Korean version (MMSE-KC), Korean version of Short Blessed test (SBT-K), Word Fluency, Korean version of Boston Naming Test (K-BNT), Word List Memory, Word List Recall, Word List Recognition, Constructional Praxis, and Constructional Recall. The subtests from the SNSB; Digit Span Forward (DSTF) and Backward (DSTB), Contrasting Program, Go/No-Go, Semantic Fluency, Phonemic Fluency, and the Stroop color test. Higher scores on all subtests, with the exception for SBT-K, indicate a better cognitive function.

### Other evaluations

Activities of Daily Living (ADL) were measured using the Seoul Instrumental Activities of Daily Living (S-IADL) [33] for AD patients and LLD patients, and Blessed Dementia Scale–Activities of Daily Living (BDS–ADL) [35] for AD patients. Behavioral disturbances of AD patients were evaluated using the Neuropsychiatric Inventory (NPI) [29]. Lower S-IADL, BDS–ADL, and NPI scores indicate better functioning. Quality of life was assessed with the Korean version of the World Health Organization-Five Well-Being Index (WHO-5) [31]. Higher scores on the WHO-5 indicate a higher level of well-being. Suicidal ideation was assessed using the Suicidal Ideation Scale (SIS) [24]. Higher scores on the SIS indicate higher suicidal ideation. Suicidality was measured using the corresponding module of the Mini-International Neuropsychiatric Interview. A suicide risk score, based on the number of items endorsed was recorded [56].

### Statistical analyses

For the descriptive statistics, a Fisher's exact test was used. To assess differences in cognitive function between groups at baseline, analysis of covariance (ANCOVA) with covariates of age, sex, and the duration of education was conducted. An independent sample *t* test was performed to compare the degree of change between the groups. Paired *t* tests were used to determine cognitive function changes in each treatment responder group. Cohen’s d (for a *t* test) and/or partial eta squared (*η*_*p*_^2^) (for an ANCOVA) were reported as a measure of effect size. The statistical analysis was performed usi148ng SPSS Statistics 23.0.

## Results

### Characteristics of sample

Table 1 shows the demographic and clinical characteristics of the entire sample. Within the AD group, there were no differences in demographic and clinical characteristics between AD + D and AD − D. Within the elderly group without dementia, the LLD and HC did not differ in age, sex and clinical characteristics. However, the educational level of the HC group was significantly higher than that of the LLD group (*p* = 0.006).

**Table 1:** Demographic characteristics and clinical features of overall group (*N* = 95)

	AD		Elderly without dementia		*p* value	
	AD + D (*n* = 29)	AD − D (*n* = 36)	LLD (*n* = 15)	HC (*n* = 15)	AD + D vs. AD − D	LLD vs. HC
Age (years, mean ± *SD*)	76.34 ± 6.81	78.86 ± 5.06	70.80 ± 5.56	74.27 ± 5.31	0.505	0.151
Sex (female/male)	24/5	26/10	13/2	13/2	0.316	1.000
Education (years, means ± SD)	3.24 ± 3.74	4.78 ± 4.44	3.27 ± 2.92	13.73 ± 16.46	0.414	0.006
*CDR*					0.281	
0.5 (*n*, %)	13 (44.8)	20 (55.6)	–	–		
1 (*n*, %)	16 (55.2)	14 (38.9)	–	–		
2 (*n*, %)	0	2 (5.6)	–	–		

All variables were compared by fisher’s exact test

AD + D, Alzheimer’s disease with depression; AD − D, Alzheimer’s disease without depression; LLD, late-life depression; HC, healthy controls; SD, standard deviation; CDR, Clinical Dementia Rating

### Comparison of cognitive function and other clinical data at baseline

Table 2 presents the differences in cognitive function between subgroups (the depressed subgroup and the non-depressed subgroup) of the AD group and the elderly group without dementia at baseline. Each subgroup with depression showed poor cognitive performance in some domains compared to the subgroup without depression. The mean score of K-BNT, which is a language function test, was lower in AD + D compared to AD − D (*F* = 5.346, *p* = 0.024, *η*_*p*_^2^ = 0.082). The mean score of MMSE-KC, which measures global cognitive function, in LLD was lower than that of HC (*F* = 4.327, *p* = 0.048, *η*_*p*_^2^ = 0.148). The LLD group had lower mean scores of Contrasting, Go/No-Go, and Semantic Fluency tests, which are executive function tests, compared to HC group (*F* = 5.346, *p* = 0.029, *η*_*p*_^2^ = 0.176, *F* = 15.980, *p* = 0.000, *η*_*p*_^2^ = 0.390, and *F* = 5.385, *p* = 0.030, *η*_*p*_^2^ = 0.197, respectively).

**Table 2:** Comparison of cognitive function and other clinical data according to group at baseline

	AD		Elderly without dementia		AD + D vs. AD − D		LLD vs. HC	
	AD + D (mean ± SD)	AD − D (mean ± SD)	LLD (mean ± SD)	HC (mean ± SD)	*p* value	Cohen’s *d*/*η*_*p*_^2^	*p* value	Cohen’s d/*η*_*p*_^2^
*Depressive symptoms*								
CSDD	14.92 ± 6.95	1.75 ± 2.05	–	–	0.000	2.57	–	–
HAMD	–	–	14.13 ± 5.48	0.20 ± 0.56	–		0.000	3.58
SGDS-K	11.55 ± 3.16	2.44 ± 2.48	9.67 ± 3.81	1.07 ± 1.58	0.000	3.21	0.000	2.95
*Cognitive function*								
MMSE-KC	16.03 ± 2.97	17.31 ± 3.58	24.33 ± 3.01	27.07 ± 2.74	0.242	0.023	0.048*	0.148
Word Fluency	6.52 ± 2.90	7.39 ± 3.37	11.07 ± 2.63	13.67 ± 2.82	0.474	0.009	0.169	0.074
K-BNT	4.76 ± 2.31	6.61 ± 3.17	9.00 ± 2.70	11.27 ± 2.43	0.024*	0.082	0.237	0.055
Word List Memory	8.00 ± 3.18	8.48 ± 3.154	16.71 ± 4.97	18.40 ± 3.36	0.550	0.008	0.287	0.047
Word List Recall	1.42 ± 1.35	1.18 ± 1.13	5.57 ± 2.03	5.40 ± 1.77	0.602	0.006	0.932	0.000
Word List Recognition	4.05 ± 3.40	4.52 ± 3.02	8.71 ± 3.22	9.07 ± 1.03	0.456	0.012	0.364	0.034
Constructional Praxis	5.93 ± 2.15	6.89 ± 2.36	8.60 ± 1.84	9.47 ± 1.81	0.358	0.014	0.706	0.006
Constructional Recall	1.14 ± 1.41	1.19 ± 1.83	3.40 ± 1.84	6.13 ± 3.38	0.736	0.002	0.262	0.050
SBT-K	20.32 ± 5.73	17.69 ± 6.72	5.07 ± 5.85	2.33 ± 2.77	0.234	0.024	0.422	0.026
DSTF	4.17 ± 0.82	4.46 ± 1.07	4.93 ± 1.34	5.80 ± 1.37	0.822	0.001	0.398	0.029
DSTB	2.21 ± 1.06	2.37 ± 1.03	2.73 ± 1.34	3.47 ± 0.92	0.845	0.001	0.135	0.087
Contrasting Program	10.08 ± 7.46	11.86 ± 7.06	17.47 ± 3.52	19.87 ± 0.35	0.491	0.009	0.029*	0.176
Go/No-Go	8.96 ± 6.61	9.69 ± 6.43	12.87 ± 6.12	19.93 ± 0.26	0.927	0.000	0.000***	0.390
Semantic Fluency	15.71 ± 4.12	14.91 ± 6.65	24.85 ± 5.46	33.43 ± 6.96	0.335	0.017	0.030*	0.197
Phonemic Fluency	7.200 ± 7.24	7.44 ± 8.14	17.08 ± 11.20	28.13 ± 10.05	0.820	0.001	0.241	0.062
Stroop color	24.08 ± 16.15	32.56 ± 18.52	62.85 ± 22.09	79.27 ± 20.40	0.251	0.040	0.325	0.042
*Others*								
S-IADL	18.00 ± 9.40	18.36 ± 8.37	4.40 ± 2.41	0.73 ± 0.96	0.872	−0.04	0.000***	2.00
BDS–ADL	2.17 ± 1.85	1.97 ± 1.41	–	–	0.623	0.12	–	–
NPI	7.36 ± 5.00	3.53 ± 3.86	–	–	0.001**	0.86	–	–
WHO-5	28.41 ± 22.46	60.03 ± 17.91	21.60 ± 17.75	66.13 ± 19.06	0.000***	−1.56	0.000***	−2.42
SIS	7.55 ± 3.10	5.08 ± 0.28	6.00 ± 2.73	4.67 ± 1.29	0.000***	1.12	0.098	0.62
Suicidality	4.83 ± 6.57	0.08 ± 0.28	2.13 ± 2.92	0.33 ± 1.29	0.001**	1.02	0.042*	0.80

The cognitive functions were compared by ANCOVA after controlling for age, sex, and educational duration. Psychosocial and other variables were compared by independent sample *t* test. Effect sizes were calculated using Cohen’s d (for an independent sample *t* test) and/or partial eta squared (*η*_*p*_^2^) (for an ANCOVA)

AD + D, Alzheimer’s disease with depression; AD − D, Alzheimer’s disease without depression; LLD, late-life depression; HC, healthy controls; SD, standard deviation; CSDD, Cornell Scale for Depression in Dementia; HAMD, Hamilton Rating Scale for Depression; SGDS-K, Short Geriatric Depression Scale-Korean version; MMSE-KC, Korean version mini-mental state examination; K-BNT, Korean version of the Boston Naming Test; SBT-K, Korean version of Short Blessed test; DSTF, Digit Span Forward; DSTB, Digit Span Backward; S-IADL, Seoul Instrumental Activities of Daily Living; BDS-ADL, Blessed Dementia Scale–Activities of Daily Living; WHO-5, Korean version of the World Health Organization-Five Well-Being Index; SIS, Suicidal Ideation Scale

*p < .05, **p < *.*01, ***p < .001

There were also significant differences in other factors between the depressed and the non-depressed, in both the AD and the elderly without dementia groups. AD + D had higher mean scores on the NPI (*t* = 3.401, *p* = 0.001, Cohen’s *d* = 0.86), SIS (*t* = 4.273, *p* = 0.000, Cohen’s *d* = 1.12), and Suicidality (*t* = 3.883, *p* = 0.001, Cohen’s *d* = 1.02) assessments and lower mean scores on the WHO-5 (*t* = −6.386, *p* = 0.000, Cohen’s *d* = −1.56) compared to AD − D. The LLD group showed higher mean scores on the S-IADL (*t* = 5.465, *p* = 0.000, Cohen’s *d* = 2.00) and Suicidality (*t* = 2.181, *p* = 0.042, Cohen’s *d* = 0.80) and lower mean scores on the WHO-5 (*t* = −6.623, *p* = 0.000, Cohen’s *d* = −2.42) compared to the HC group.

### Comparison of changes in cognitive function at 16 weeks

**Fig. 1:**
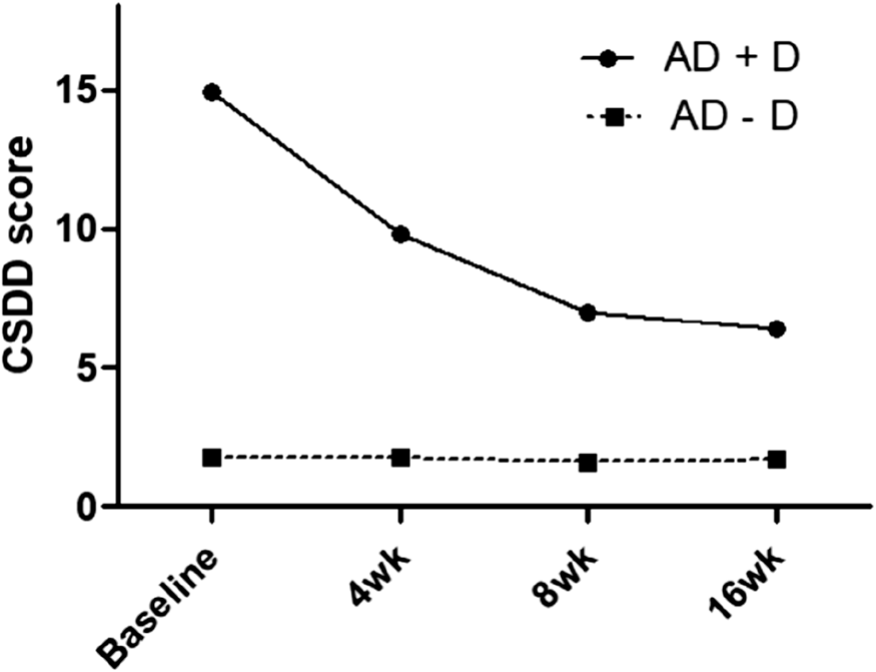
Cornell scale for depression (CSDD) mean score changes in both Alzheimer’s disease (AD) groups during 16 weeks of treatment

To determine the effect of depression treatment on clinical factors, we first analyzed whether depressive symptoms were significantly reduced after 16 weeks in AD + D and LLD subgroups with depression. In both AD + D and LLD, the mean score of depressive symptoms (as measured by CSDD for AD + D or HAMD for LLD) within each subgroup was significantly diminished from baseline to final (*t* = 6.807, *p* = 0.000, Cohen’s *d* = 1.605 and *t* = 8.639, *p* = 0.000, Cohen’s *d* = 2.396, respectively). Figure 1 indicates CSDD mean score changes during 16 weeks of treatment in both AD groups. In addition, the mean score of SGDS-K in both groups (i.e., AD + D and LLD) was significantly reduced (*t* = 5.684, *p* = 0.000, Cohen’s *d* = 1.340 and *t* = 8.215, *p* = 0.000, Cohen’s *d* = 2.279, respectively).

**Fig. 2:**
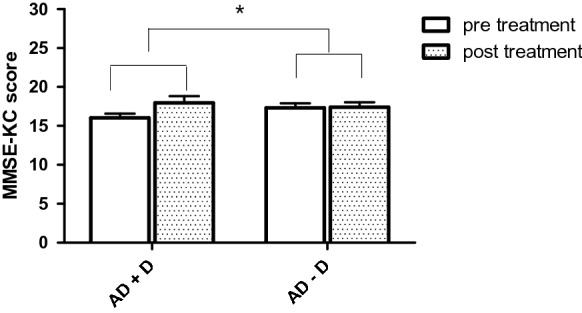
AD + D group exhibited significantly greater increase on mean change score of MMSE-KC than AD − D (*t* = 2.169, *p* = 0.034). The bars indicate standard deviations

**Fig. 3:**
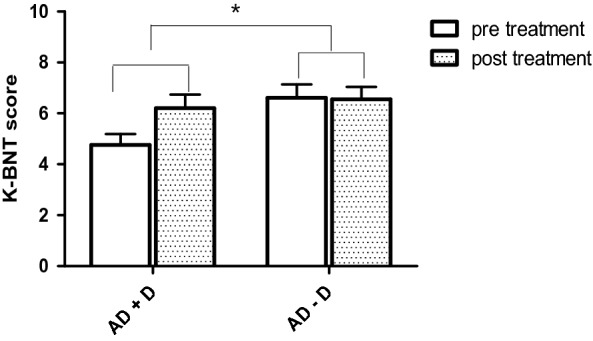
After depression treatment, the AD + D group showed greater significant improvement in mean change score of K-BNT compared with AD − D group (*t* = 2.945, *p* = 0.005). The bars indicate standard deviations

Table 3 presents a comparison of the mean scores on cognitive changes from baseline to final measurement for all neuropsychological tests between AD + D and AD − D or LLD and HC. The AD + D group showed a greater improvement in scores on the MMSE-KC (Fig. 2) and K-BNT (Fig. 3) than the AD − D group (*t* = 2.169, *p* = 0.034, Cohen’s *d* = 0.542 and *t* = 2.945, *p* = 0.005, Cohen’s *d* = 0.756, respectively). The LLD group showed a significant improvement on the Go/No-Go test as compared to the HC group (*t* = 3.144, *p* = 0.007, Cohen’s *d* = 1.147).

**Table 3:** Comparison of changes in clinical variables between pre- and post-treatment

	AD		Elderly without dementia		AD + D vs. AD − D		LLD vs. HC	
	AD + D (mean ± SD)	AD − D (mean ± SD)	LLD (mean ± SD)	HC (mean ± SD)	*p* value	Cohen’s *d*	*p* value	Cohen’s *d*
*Depressive symptoms*								
CSDD	8.15 ± 7.46	0.06 ± 2.28	–	–	0.000***	1.467	–	–
HAMD	–	–	11.20 ± 7.24	−0.20 ± 1.08	–	–	0.000***	2.202
SGDS-K	3.76 ± 4.60	−1.25 ± 4.42	6.54 ± 4.29	−0.86 ± 2.18	0.000***	1.110	0.000***	2.175
*Cognitive function*								
MMSE-KC	1.93 ± 3.37	0.08 ± 3.45	0.73 ± 3.08	0.13 ± 2.03	0.034*	0.542	0.534	0.235
Word Fluency	1.24 ± 3.17	0.60 ± 3.27	1.15 ± 2.48	1.00 ± 2.50	0.431	0.199	0.870	0.060
K-BNT	1.48 ± 2.50	−0.06 ± 1.43	0.00 ± 2.48	0.13 ± 1.30	0.005**	0.756	0.864	0.065
Word List Memory	2.26 ± 5.44	1.16 ± 3.40	1.17 ± 2.95	1.07 ± 3.73	0.374	0.242	0.940	0.029
Word List Recall	0.95 ± 1.68	0.28 ± 1.30	0.50 ± 1.78	1.13 ± 1.73	0.120	0.446	0.360	0.358
Word List Recognition	1.67 ± 4.19	0.47 ± 3.41	0.83 ± 2.33	0.53 ± 0.83	0.278	0.314	0.646	0.171
Constructional Praxis	0.28 ± 1.67	0.67 ± 1.99	−0.08 ± 1.55	0.07 ± 1.44	0.401	0.212	0.801	0.100
Constructional Recall	0.04 ± 1.43	−0.50 ± 1.78	1.62 ± 3.25	0.60 ± 3.27	0.199	0.334	0.419	0.312
SBT-K	2.25 ± 5.64	−0.31 ± 5.88	0.54 ± 5.93	1.27 ± 2.22	0.084	0.444	0.662	0.163
DSTF	0.04 ± 0.91	0.34 ± 0.91	−0.08 ± 0.95	0.13 ± 1.06	0.215	0.329	0.588	0.208
DSTB	−0.04 ± 0.62	0.17 ± 0.95	−0.15 ± 0.80	0.13 ± 1.30	0.341	0.261	0.497	0.304
Contrasting Program	2.46 ± 7.92	3.06 ± 5.66	1.69 ± 3.25	0.13 ± 0.35	0.736	0.087	0.110	0.674
Go/No-Go	2.08 ± 7.92	0.63 ± 5.67	4.33 ± 5.96	−0.60 ± 1.18	0.414	0.210	0.007**	1.147
Semantic Fluency	2.00 ± 5.53	0.70 ± 5.10	2.25 ± 3.72	2.43 ± 4.03	0.362	0.244	0.908	0.046
Phonemic Fluency	2.47 ± 5.04	2.03 ± 4.12	1.92 ± 7.28	0.67 ± 5.88	0.757	0.095	0.625	0.188
Stroop color	5.54 ± 12.49	6.08 ± 12.22	8.82 ± 19.91	−0.43 ± 9.00	0.889	0.043	0.134	0.598
*Others*								
S-IADL	0.14 ± 7.92	−0.39 ± 7.75	3.00 ± 2.97	−0.07 ± 1.14	0.788	0.07	0.003**	1.36
BDS–ADL	−0.09 ± 1.75	−0.55 ± 1.71	–	–	0.300	0.27	–	–
NPI	2.82 ± 4.80	0.85 ± 5.61	–	–	0.148	0.38	–	–
WHO-5	2.90 ± 28.04	−2.91 ± 22.38	34.93 ± 20.08	4.29 ± 19.87	0.360	0.23	0.000***	1.53
SIS	2.90 ± 3.08	0.08 ± 0.28	1.33 ± 3.24	−0.36 ± 1.34	0.000***	1.29	0.080	0.68
Suicidality	3.69 ± 5.27	0.03 ± 0.38	1.33 ± 3.06	0.36 ± 1.34	0.001**	0.98	0.270	0.41

The cognitive function, psychosocial and other variables were compared by Independent sample *t* test. Effect sizes were calculated using Cohen’s *d*

AD + D, Alzheimer’s disease with depression; AD − D, Alzheimer’s disease without depression; LLD, late-life depression; HC, healthy controls; SD, standard deviation; CSDD, Cornell Scale for Depression in Dementia; HAMD, Hamilton Rating Scale for Depression; SGDS-K, Short Geriatric Depression Scale-Korean version; MMSE-KC, Korean version mini-mental state examination; K-BNT, Korean version of the Boston Naming Test; SBT-K, Korean version of Short Blessed test; DSTF, Digit Span Forward; DSTB, Digit Span Backward; S-IADL, Seoul Instrumental Activities of Daily Living; BDS-ADL, Blessed Dementia Scale–Activities of Daily Living; WHO-5, Korean version of the World Health Organization-Five Well-Being Index; SIS, Suicidal Ideation Scale

*p < .05, **p < .01, ***p < .001

### Comparison of cognitive changes between pre- and post- treatment according to the depression treatment responder group

The response rate of depression treatment was 62.07% (18 of 29) in AD + D (defined as a ≥ 50% reduction in CSDD score) and 86.67% (13 of 15) in LLD (defined as a ≥ 50% reduction in HAMD score). At 16 weeks, we examined the changes in cognitive function before and after treatment according to the response to depression treatment to confirm whether the improvements in cognitive function in some domains were indeed due to the treatment effect of the medication.

Table 4 indicates the comparison of mean changes in cognitive function before and after treatment within the depression treatment responder group in AD + D and LLD, respectively. The responders to depression treatment in AD + D showed a significant improvement in scores on the MMSE-KC (*t* = −2.317, *p* = 0.033, Cohen’s *d* = −0.546) and the K-BNT (*t* = −2.197, *p* = 0.042, Cohen’s *d* = −0.518). The responders to depression treatment in the LLD group showed a significant improvement in the MMSE-KC (*t* = −2.379, *p* = 0.035, Cohen’s *d* = −0.660) and Go/No-Go test (*t* = −2.264, *p* = 0.043, Cohen’s *d* = −0.628). In contrast, in non-responders to depression treatment of the two groups (AD + D and LLD), there was no difference in pre- and post-treatment scores in any cognitive domains.

**Table 4:** Comparison of changes in clinical variables between pre- and post-treatment in depression treatment responder groups

	Responders in AD + D (*n* = 18)				Responders in LLD (*n* = 13)			
	Baseline (mean ± SD)	After 16 weeks (mean ± SD)	*p* value	Cohen’s *d*	Baseline (mean ± SD)	After 16 weeks (mean ± SD)	*p* value	Cohen’s *d*
*Depressive Symptoms*								
CSDD	14.44 ± 7.58	3.50 ± 3.13	0.000**	1.605	–	–	–	–
HAMD	–	–	–		14.54 ± 5.62	1.38 ± 1.26	0.000***	2.396
SGDS-K	11.11 ± 3.69	5.61 ± 0.88	0.000***	1.340	9.69 ± 4.11	2.00 ± 1.78	0.000***	2.279
*Cognitive function*								
MMSE-KC	16.28 ± 3.29	18.28 ± 4.75	0.033*	−0.546	24.15 ± 3.21	25.69 ± 3.66	0.035*	−0.660
Word Fluency	7.28 ± 2.82	8.33 ± 3.53	0.138	−0.367	11.15 ± 2.76	12.69 ± 3.38	0.052	−0.599
K-BNT	4.67 ± 2.38	6.06 ± 2.96	0.042*	−0.518	9.45 ± 2.95	10.00 ± 3.52	0.441	−0.242
Word List Memory	7.60 ± 3.24	8.80 ± 3.19	0.493	−0.226	17.40 ± 5.46	18.10 ± 5.20	0.472	−0.238
Word List Recall	1.10 ± 0.88	1.80 ± 1.14	0.132	−0.523	6.10 ± 1.79	6.60 ± 1.65	0.440	−0.255
Word List Recognition	4.67 ± 2.92	5.00 ± 3.35	0.818	−0.079	9.70 ± 0.68	9.90 ± 0.32	0.343	−0.316
Constructional Praxis	5.78 ± 2.44	5.94 ± 2.31	0.687	−0.097	8.55 ± 1.86	8.73 ± 2.49	0.676	−0.130
Constructional Recall	1.06 ± 1.48	0.82 ± 1.85	0.509	0.164	3.45 ± 2.02	5.27 ± 2.72	0.101	−0.544
SBT-K	20.18 ± 5.87	18.88 ± 7.38	0.165	0.353	4.92 ± 6.09	5.23 ± 5.45	0.843	−0.056
DSTF	4.29 ± 0.85	4.41 ± 0.94	0.608	−0.127	4.85 ± 1.28	4.85 ± 1.07	1.000	0.000
DSTB	2.24 ± 1.09	2.18 ± 0.95	0.718	0.089	2.62 ± 1.39	2.77 ± 1.09	0.502	−0.192
Contrasting Program	9.12 ± 7.43	10.82 ± 8.01	0.376	−0.221	17.77 ± 3.56	19.00 ± 2.74	0.143	−0.434
Go/No-Go	9.00 ± 6.86	9.76 ± 5.72	0.685	−0.100	14.00 ± 5.46	17.00 ± 3.27	0.043*	−0.628
Semantic Fluency	16.06 ± 4.29	18.35 ± 7.87	0.114	−0.405	25.50 ± 4.99	27.00 ± 5.66	0.160	−0.484
Phonemic Fluency	7.90 ± 8.36	10.60 ± 10.30	0.056	−0.694	20.56 ± 10.48	22.11 ± 13.69	0.590	−0.187
Stroop color	25.44 ± 15.65	34.56 ± 17.34	0.062	−0.721	61.58 ± 22.58	66.08 ± 30.30	0.604	−0.154
*Others*								
S-IADL	17.22 ± 8.82	16.78 ± 9.58	0.833	0.050	4.23 ± 1.79	2.08 ± 2.25	0.005**	0.950
BDS–ADL	1.72 ± 1.22	1.97 ± 1.34	0.535	−0.149	–	–	–	–
NPI	6.17 ± 4.13	3.22 ± 5.01	0.005**	0.769	–	–	–	–
WHO-5	31.78 ± 26.48	40.67 ± 15.78	0.243	−0.285	21.23 ± 18.86	60.62 ± 15.99	0.000***	−2.301
SIS	6.67 ± 2.57	4.50 ± 2.01	0.004**	0.794	6.00 ± 2.92	4.62 ± 1.39	0.177	0.398
Suicidality	4.56 ± 8.17	1.33 ± 3.17	0.045*	0.510	2.38 ± 3.07	0.85 ± 1.73	0.114	0.472

The cognitive function, psychosocial and other variables were compared by Paired *t* test. Effect sizes were calculated using Cohen's *d*

AD + D, Alzheimer’s disease with depression; AD − D, Alzheimer’s disease without depression; LLD, late-life depression; HC, healthy controls; SD, standard deviation; CSDD, Cornell Scale for Depression in Dementia; HAMD, Hamilton Rating Scale for Depression; SGDS-K, Short Geriatric Depression Scale-Korean version; MMSE-KC, Korean version mini-mental state examination; K-BNT, Korean version of the Boston Naming Test; SBT-K, Korean version of Short Blessed test; DSTF, Digit Span Forward; DSTB, Digit Span Backward; S-IADL, Seoul Instrumental Activities of Daily Living; BDS-ADL, Blessed Dementia Scale–Activities of Daily Living; WHO-5, Korean version of the World Health Organization-Five Well-Being Index; SIS, Suicidal Ideation Scale

*p < .05, **p < .01, ***p < .001

### Comparison of changes others clinical data at 16 weeks

As shown in Table 3, after 16 weeks, AD + D showed a decrease in scores in SIS (*t* = 4.911, *p* = 0.000, Cohen’s *d* = 1.29) and Suicidality (*t* = 3.733, *p* = 0.001, Cohen’s *d* = 0.98) compared to AD − D. The responders to depression treatment in AD + D showed a significant decrease in scores on the NPI (*t* = 3.264, *p* = 0.005, Cohen’s *d* = 0.769), SIS (*t* = 3.370, *p* = 0.004, Cohen’s *d* = 0.794), and Suicidality (*t* = 2.163, *p* = 0.045, Cohen’s *d* = 0.510) (Table 4). The LLD group showed a decrease in scores on the S-IADL (*t* = 3.495, *p* = 0.003, Cohen’s *d* = 1.36) and an improvement in scores on the WHO-5 (*t* = 4.127, *p* = 0.000, Cohen’s *d* = 1.53) (Table 3). The similar results are presented in the analysis within depression treatment responders in LLD (Table 4).

## Discussion

In this study, the difference in cognitive function between AD + D and AD − D or LLD and HC was examined, and cognitive domains in response to depression treatment were measured using a comprehensive neuropsychological test battery.

At the baseline assessment, the subgroups with depression (i.e., AD + D or LLD) showed greater deficit in some domains, such as language function in AD or executive function in LLD, than those without depression (i.e., AD − D or HC) (Table 2). AD + D had greater impairment in language function (as measured by K-BNT) than AD − D. LLD showed more deficit in global cognitive function (as measured by MMSE-KC) and executive function (as measured by contrasting program, Go/No-Go, and semantic word fluency test) than HC. These cognitive differences could not be explained through a cross-sectional comparison. Hence, we observed changes in cognitive function after the antidepressant treatment. We found that more impaired cognitive domains (i.e., language function for AD + D or executive function for LLD) at baseline in subgroups with depression were improved compared to those without depression, respectively (Table 3), following the depression treatment. We also identified similar results in the analysis within the depression treatment responders of two subgroups with depression for each group (Table 4), which means that these outcomes were the result of cognitive alterations from a real effect of depression recovery. Furthermore, two depression treatment response groups showed improvement on global cognitive function (Table 4), whereas the treatment non-response groups did not show cognitive change in any domains. Our findings suggest that depression may lead to a decline in cognitive function, and successful treatment of depression can recover cognitive impairment in AD and in the elderly population without dementia.

Previous studies found that depression impacts the cognitive progression of AD [60] and that depression treatment enhances global cognitive function in AD [52, 63]. However, in these studies, it was not specified which cognitive domains were related to depression, because a brief cognitive screening tool, MMSE, was used to assess cognitive functioning. In the current study, we identified more specific domain, such as language function, that are impacted by depression in AD + D. The Boston Naming Test (BNT), one of the subtests used to measure language function in our study, is an instrument to assess confrontation naming ability, and it is commonly used to measure language function in AD patients [10]. Language impairment on the BNT is one of the primary determinants of cognitive decline in AD [19] and a common symptom among individuals with dementia [62]. The language impairment is often found in the early stage of AD and deteriorates over the course of disease [58, 65].

At baseline, in our study, the AD + D group had more impaired language function than the AD − D group, but it was unclear whether language dysfunction was caused by neurodegenerative changes or temporarily damaged due to depression. After depression treatment, a significant improvement in language function was observed in AD + D compared to AD − D; hence, it is plausible that some degree of language impairment in AD + D stems from depression. This finding implies that language function in AD might be vulnerable to depression, which could contribute to poor prognosis of AD. Despite the high prevalence of depression in AD, the evaluation and treatment of depression in clinical settings is often overlooked. Our findings suggest the importance of evaluation and treatment of depression in AD patients, since comorbid depression in AD could contribute to a decline in cognitive functioning.

Cognitive domain influenced by depression in AD + D was different from those with LLD. It is presumed that regions of brain affected by depression may differ between AD and LLD. In AD, the medial temporal lobe of the brain is the first area to exhibit atrophy [14, 20, 27, 30], which is characterized by the most extensive pathological change in AD [59]. Depression is known to be neurotoxic to medial temporal lobe structures and can contribute to their atrophy [16, 42, 57]. There is evidence for significant effects of depressive symptoms on medial temporal lobe structure in AD patients with depressive symptoms [15, 17]. In our study, AD patients with depression showed greater naming disturbance. It is well-known that naming difficulty is associated with lesions in the medial temporal lobe [12, 23]. It could be postulated that depression in AD might aggravate atrophy of the medial temporal lobe, which may in turn lead to more impaired language function. It is inferred that if depression is not adequately treated in AD, the medial temporal lobe atrophy might become worse, causing poorer prognosis of disease.

Also, in LLD, we identified depression may impact the executive function. There are previous reports of frontal lobe abnormalities in LLD [2, 54]. In this respect, it is likely that depression’s impact on the regions of brain is different in AD from in LLD. To clary determine, longitudinal studies on structural changes in the brain following depression treatment may be helpful.

AD + D had lower response rates to depression treatment than LLD (AD + D, 62.07%, LLD 86.67%) (Table 4), which is consistent with findings of a previous study [44]. In a meta-analysis study, the efficacy of selective serotonin reuptake inhibitors (SSRIs) treatment in AD + D appeared to be quite weak [55], while SSRIs are generally considered to be as the first choice antidepressant for LLD. In our study, low levels of antidepressant response rate in AD + D compared to LLD could be evidence supporting the concept that depression in AD has a different pathogenic mechanism from depression in the elderly with normal cognition [9, 11].

Overall, this study demonstrated that the patterns of depression seen in AD differed from typical depression seen in the elderly without dementia, specifically regarding the affected cognitive domains and antidepressant response rate. From our results, depression in AD might be thought of as a different subtype of depression. Future studies should explore the possibility of differences in pathophysiology between AD + D and LLD to determine whether depression in AD patients requires a different therapeutic approach. Moreover, this study demonstrated that depression treatment had beneficial effects on decreasing behavioral disturbances and suicide-related factors in AD patients with depression, and improving ADL and QOL in the elderly with LLD (Tables 3, 4).

This study had several strengths. We identified language impairment as a specific cognitive domain impacted by depression in those with AD, which improved through the treatment of depression via SSRIs. This is the first study to examine the specific cognitive domains impacted by AD and comorbid depression. We also found that a different cognitive function, specifically executive function, are affected in patients with late-life depression, but without dementia. Our research thus suggests that different therapeutic approaches may be required when treating depression in AD patients, compared to those required for treating patients without dementia. Our study, could consider the low antidepressant response rates in the AD patients with depression compared to those in patients with late-life depression, to support the concept that the pathogenic mechanism of AD-related depression could be different from depression in the elderly with normal cognition.

Our study had a few limitations. The small sample size might have limited the generalizability of the results. In the future, studies with larger samples are needed to determine the effect of depression treatment on cognitive outcomes in AD patients. Another limitation was that the healthy control group had a significantly higher level of education than that of the LLD group. Although we statistically adjusted for age, sex, and education when analyzing cognitive function between the groups, considerable differences in the level of education might actually have influenced the psychometric assessment.

Nevertheless, the current study has demonstrated the effectiveness of treatment for depression in AD patients as well as elderly population without dementia regarding improvement in cognitive functioning (language and executive functioning). This is valuable as a frontier study in examining of the association between depression treatment and cognitive outcomes in AD.

In conclusion, our study demonstrated that depression treatment positively influences not only cognitive function but also overall functioning and other psychosocial factors in AD and LLD. Our findings have important clinical implication for diagnosis and treatment of depression in both AD patients and the elderly population without dementia.

## Data Availability

The data sets analyzed in the current study are available from the corresponding author on reasonable request.

## Abbreviations

AD + D: Alzheimer’s disease with depression
AD − D: Alzheimer’s disease without depression
LLD: Late-life depression
HC: Healthy controls
AChEIs: Acetylcholinesterase inhibitors
SSRIs: Selective serotonin reuptake inhibitors
CSDD: Cornell Scale for Depression in Dementia
HAMD: Hamilton Rating Scale for Depression
SGDS-K: Short Geriatric Depression Scale-Korean version
MMSE-KC: Korean version mini-mental state examination
K-BNT: Korean version of the Boston Naming Test
SBT-K: Korean version of Short Blessed test
DSTF: Digit Span Forward
DSTB: Digit Span Backward
S-IADL: Seoul Instrumental Activities of Daily Living
BDS–ADL: Blessed Dementia Scale–Activities of Daily Living
WHO-5: Korean version of the World Health Organization-Five Well-Being Index
SIS: Suicidal Ideation Scale

## References

[1] Alexopoulos GS, Abrams RC, Young RC, Shamoian CA (1988). Cornell scale for depression in dementia. Biol Psychiat.

[2] Almeida O, Burton E, Ferrier N, McKeith I (2003). Depression with late onset is associated with right frontal lobe atrophy. Psychol Med.

[3] Araujo NB, Moraes HS, Silveira H, Arcoverde C, Vasques PE, Barca ML (2014). Impaired cognition in depression and Alzheimer (AD): a gradient from depression to depression in AD. Arq Neuropsiquiatr.

[4] American Psychiatric Association (2013). Diagnostic and statistical manual of mental disorders.

[5] American Psychiatric Publication (2013). Diagnostic and statistical manual of mental disorders (DSM-5^®^).

[6] Bae JN, Cho MJ (2004). Development of the Korean version of the Geriatric Depression Scale and its short form among elderly psychiatric patients. J Psychosom Res.

[7] Bak TH, Mioshi E (2007). A cognitive bedside assessment beyond the MMSE: the Addenbrooke’s Cognitive Examination. Pract Neurol.

[8] Balsamo M, Cataldi F, Carlucci L, Padulo C, Fairfield B (2018). Assessment of late-life depression via self-report measures: a review. Clin Interv Aging.

[9] Banerjee S, Hellier J, Dewey M, Romeo R, Ballard C, Baldwin R (2011). Sertraline or mirtazapine for depression in dementia (HTA-SADD): a randomised, multicentre, double-blind, placebo-controlled trial. The Lancet.

[10] Baum CM, Edwards DF, Leavitt K, Grant E, Deuel RM (1988). Performance components in senile dementia of the Alzheimer type: motor planning, language, and memory. Occup Therapy J Res.

[11] Brodaty H (2011). Antidepressant treatment in Alzheimer's disease. The Lancet.

[12] Busch RM, Frazier TW, Haggerty KA, Kubu CS (2005). Utility of the Boston Naming Test in predicting ultimate side of surgery in patients with medically intractable temporal lobe epilepsy. Epilepsia.

[13] Butters MA, Whyte EM, Nebes RD, Begley AE, Dew MA, Mulsant BH (2004). The nature and determinants of neuropsychological functioning in late-lifedepression. Arch Gen Psychiatry.

[14] De Leon M, George A, Stylopoulos L, Smith G, Miller D (1989). Early marker for Alzheimer's disease: the atrophic hippocampus. The Lancet.

[15] Dhikav V, Sethi M, Anand K (2014). Medial temporal lobe atrophy in Alzheimer's disease/mild cognitive impairment with depression. Br J Radiol.

[16] Eker C, Gonul AS (2010). Volumetric MRI studies of the hippocampus in major depressive disorder: meanings of inconsistency and directions for future research.

[17] Enache D, Cavallin L, Lindberg O, Farahmand B, Kramberger MG, Westman E (2015). Medial temporal lobe atrophy and depressive symptoms in elderly patients with and without Alzheimer disease. J Geriatr Psychiatry Neurol.

[18] Fahlander K, Berger A-K, Wahlin Å, Bäckman L (1999). Depression does not aggravate the episodic memory deficits associated with Alzheimer's disease. Neuropsychology.

[19] Ferris SH, Farlow M (2013). Language impairment in Alzheimer’s disease and benefits of acetylcholinesterase inhibitors. Clin Interv Aging.

[20] Fox N, Warrington E, Freeborough P, Hartikainen P, Kennedy A, Stevens J, Rossor MN (1996). Presymptomatic hippocampal atrophy in Alzheimer's disease: a longitudinal MRI study. Brain.

[21] Gallassi R, Morreale A, Pagni P (2001). The relationship between depression and cognition. Arch Gerontol Geriatr.

[22] Gonzales-Salvdor T, Lyketsos C, Baker A, Roques C, Hovanek L, Steele C (2000). Quality of life of patients with dementia in long-term care. Int J Geriatr Psychiatry.

[23] Goodglass H, Wingfield A (1997). Anomia: neuroanatomical and cognitive correlates.

[24] Harlow LL, Newcomb MD, Bentler PM (1986). Depression, self-derogation, substance use, and suicide ideation: Lack of purpose in life as a mediational factor. J Clin Psychol.

[25] Hofman M, Seifritz E, Kräuchi K, Hock C, Hampel H, Neugebauer A, Müller-Spahn F (2000). Alzheimer's disease, depression and normal ageing: merit of simple psychomotor and visuospatial tasks. Int J Geriatr Psychiatry.

[26] Hughes CP, Berg L, Danziger W, Coben LA, Martin RL (1982). A new clinical scale for the staging of dementia. Br J Psychiatry.

[27] Juottonen K, Laakso M, Insausti R, Lehtovirta M, Pitkänen A, Partanen K, Soininen H (1998). Volumes of the entorhinal and perirhinal cortices in Alzheimer’s disease. Neurobiol Aging.

[28] Kang Y, Na D, Hahn S (2003). Seoul neuropsychological screening battery.

[29] Kaufer DI, Cummings JL, Ketchel P, Smith V, MacMillan A, Shelley T (2000). Validation of the NPI-Q, a brief clinical form of the Neuropsychiatric Inventory. J Neuropsychiatry Clin Neurosci.

[30] Killiany RJ, Moss MB, Albert MS, Sandor T, Tieman J, Jolesz F (1993). Temporal lobe regions on magnetic resonance imaging identify patients with early Alzheimer's disease. Arch Neurol.

[31] Kim HJ, Moon YS, Son BK, Lee SK, Rho HJ, Kim DH (2010). The utility of Korean version of the WHO five well-being index in evaluating depressive symptoms and quality of life in the aged dwelling in community. J Korean Geriatr Psychiatry.

[32] Koenig AM, DeLozier IJ, Zmuda MD, Marron MM, Begley AE, Anderson SJ (2015). Neuropsychological functioning in the acute and remitted states of late-life depression. J Alzheimers Dis.

[33] Ku HM, Kim JH, Kwon EJ, Kim SH, Lee HS, Ko HJ (2004). A study on the reliability and validity of Seoul-Instrumental Activities of Daily Living (S-IADL). J Korean Neuropsychiatr Assoc.

[34] Kuzis G, Sabe L, Tiberti C, Dorrego F, Starkstein S (1999). Neuropsychological correlates of apathy and depression in patients with dementia. Neurology.

[35] Lee JH, Lee KU, Lee DY, Kim KW, Jhoo JH, Kim JH (2002). Development of the Korean Version of the Consortium to Establish a Registry for Alzheimer's Disease Assessment Packet (CERAD-K) clinical and neuropsychological assessment batteries. J Gerontol B Psychol Sci Soc Sci.

[36] Leong C (2014). Antidepressants for depression in patients with dementia: a review of the literature. Consult Pharm.

[37] Lichtenberg PA, Steiner DA, Marcopulos BA, Tabscott JA (1992). Comparison of the Hamilton Depression Rating Scale and the Geriatric Depression Scale: detection of depression in dementia patients. Psychol Rep.

[38] Lopez OL, Boller F, Becker JT, Miller M, Reynolds CF (1990). Alzheimer's disease and depression: neuropsychological impairment and progression of the illness. Am J Psychiatry.

[39] Lozupone M, La Montagna M, D’Urso F, Piccininni C, Sardone R, Dibello V (2018). Pharmacotherapy for the treatment of depression in patients with alzheimer’s disease: a treatment-resistant depressive disorder. Expert Opin Pharmacother.

[40] Lyketsos CG, DelCampo L, Steinberg M, Miles Q, Steele CD, Munro C (2003). Treating depression in Alzheimer disease: efficacy and safety of sertraline therapy, and the benefits of depression reduction: the DIADS. Arch Gen Psychiatry.

[41] Lyketsos CG, Steele C, Galik E, Rosenblatt A, Steinberg M, Warren A, Sheppard J-M (1999). Physical aggression in dementia patients and its relationship to depression. Am J Psychiatry.

[42] McKinnon MC, Yucel K, Nazarov A, MacQueen GM (2009). A meta-analysis examining clinical predictors of hippocampal volume in patients with major depressive disorder. J Psychiatry Neurosci.

[43] Munro CA, Longmire CF, Drye LT, Martin BK, Frangakis CE, Meinert CL (2012). Cognitive outcomes after sertaline treatment in patients with depression of Alzheimer disease. Am J Geriatr Psychiatry.

[44] Nelson JC, Delucchi K, Schneider LS (2008). Efficacy of second generation antidepressants in late-life depression: a meta-analysis of the evidence. Am J Geriatr Psychiatry.

[45] Nelson JC, Devanand DP (2011). A systematic review and meta-analysis of placebo-controlled antidepressant studies in people with depression and dementia: [See Editorial Comments by Eric J. Lenze, MD on pp 0000–0000]. J Am Geriatr Soc.

[46] Olin JT, Schneider LS, Katz IR, Meyers BS, Alexopoulos GS, Breitner JC (2002). Provisional diagnostic criteria for depression of Alzheimer disease. Am J Geriatr Psychiatry.

[47] Orgeta V, Tabet N, Nilforooshan R, Howard R (2017). Efficacy of antidepressants for depression in Alzheimer’s disease: systematic review and meta-analysis. J Alzheimers Dis.

[48] Rapp MA, Schnaider-Beeri M, Purohit DP, Perl DP, Haroutunian V, Sano M (2008). Increased neurofibrillary tangles in patients with Alzheimer disease with comorbid depression. Am J Geriatr Psychiatry.

[49] Reifler BV, Teri L, Raskind M, Veith R, Barnes R, White E, McLean P (1989). Double-blind trial of imipramine in Alzheimer’s disease patients with and without depression. Am J Psychiatry.

[50] Richard E, Reitz C, Honig LH, Schupf N, Tang MX, Manly JJ (2013). Late-life depression, mild cognitive impairment, and dementia. JAMA Neurol.

[51] Roselli F, Tartaglione B, Federico F, Lepore V, Defazio G, Livrea P (2009). Rate of MMSE score change in Alzheimer's disease: influence of education and vascular risk factors. Clin Neurol Neurosurg.

[52] Rozzini L, Chilovi BV, Conti M, Bertoletti E, Zanetti M, Trabucchi M, Padovani A (2010). Efficacy of SSRIs on cognition of Alzheimer's disease patients treated with cholinesterase inhibitors. Int Psychogeriatr.

[53] Rubio A, Vestner AL, Stewart JM, Forbes NT, Conwell Y, Cox C (2001). Suicide and Alzheimer’s pathology in the elderly: a case–control study. Biol Psychiat.

[54] Schweitzer I, Tuckwell V, Ames D (2001). Structural neuroimaging studies in late-life depression: a review. World J Biol Psychiatry.

[55] Sepehry AA, Lee PE, Hsiung GYR, Beattie BL, Jacova C (2012). Effect of selective serotonin reuptake inhibitors in Alzheimer’s disease with comorbid depression. Drugs Aging.

[56] Sheehan DV, Lecrubier Y, Sheehan KH, Amorim P, Janavs J, Weiller E (1998). The Mini-International Neuropsychiatric Interview (MINI): the development and validation of a structured diagnostic psychiatric interview for DSM-IV and ICD-10. J Clin Psychiatry.

[57] Sheline YI, Wang PW, Gado MH, Csernansky JG, Vannier MW (1996). Hippocampal atrophy in recurrent major depression. Proc Natl Acad Sci.

[58] Small JA, Sandhu N (2008). Episodic and semantic memory influences on picture naming in Alzheimer’s disease. Brain Lang.

[59] Smith A, Jobst K, Szatmari M, Jaskowski A, King E, Smith A (1992). Detection in life of confirmed Alzheimer's disease using a simple measurement of medial temporal lobe atrophy by computed tomography. Lancet.

[60] Spalletta G, Caltagirone C, Girardi P, Gianni W, Casini AR, Palmer K (2012). The role of persistent and incident major depression on rate of cognitive deterioration in newly diagnosed Alzheimer's disease patients. Psychiatry Res.

[61] Starkstein SE, Mizrahi R, Power BD (2008). Depression in Alzheimer's disease: phenomenology, clinical correlates and treatment. Int Rev Psychiatry.

[62] Tang-Wai DF, Graham NL (2008). Assessment of language function in dementia. Geriatrics.

[63] Taragano FE, Lyketsos CG, Mangone CA, Allegri RF, Comesaña-Diaz E (1997). A double-blind, randomized, fixed-dose trial of fluoxetine vs. amitriptyline in the treatment of major depression complicating Alzheimer's disease. Psychosomatics.

[64] Veiel HO (1997). A preliminary profile of neuropsychological deficits associated with major depression. J Clin Exp Neuropsychol.

[65] Vogel A, Gade A, Stokholm J, Waldemar G (2005). Semantic memory impairment in the earliest phases of Alzheimer’s disease. Dement Geriatr Cogn Disord.

[66] Wefel JS, Hoyt BD, Massman PJ (1999). Neuropsychological functioning in depressed versus nondepressed participants with Alzheimer's disease. Clin Neuropsychol.

[67] Weisenbach SL, Boore LA, Kales HC (2012). Depression and cognitive impairment in older adults. Curr Psychiatry Rep.

[68] Yi JS, Bae SO, Ahn YM, Park DB, Noh KS, Shin HK (2005). Validity and reliability of the Korean version of the Hamilton Depression Rating Scale (K-HDRS). J Korean Neuropsychiatr Assoc.

[69] Zakzanis KK, Leach L, Kaplan E (1998). On the nature and pattern of neurocognitive function in major depressive disorder. Neuropsychiatry Neuropsychol Behav Neurol.

